# Comparing closed versus open lateral internal sphincterotomy for management of chronic anal fissure: systematic review and meta-analysis of randomised control trials

**DOI:** 10.1038/s41598-023-48286-z

**Published:** 2023-11-28

**Authors:** Zelalem Asefa, Atalel Fentahun Awedew

**Affiliations:** Department of Surgery, SoM, Addis Ababa University, Addis Ababa, Ethiopia

**Keywords:** Gastroenterology, Medical research

## Abstract

Chronic anal fissure is one of the most common benign anorectal health conditions, causing significant morbidity, quality of life, and economic loss. Eight randomized controlled trials with a total population size of 1035 were eligible for analysis. Seven studies included both males and female, while one only included females. The majority of randomized controlled trials involved female dominance [54.9% (43.5–66.3)] and posterior midline location [86.1% (95% CI 81.5–90.8%)]. This meta-analysis of randomised control trials found that overall postoperative healing was 90.2%, recurrent anal fissure was 3.7%, and postoperative incontinence was 8.9% after LIS. Even though there was no statistically significant difference, closed lateral internal sphincterotomy (LIS) had higher rates of recurrent anal fissure (RR = 1.73 (95% CI 0.86–3.47, *p* = 0.90, I2 = 0%) and lower rates of postoperative incontinence rate (RR = 0.60 (95% CI 0.37–0.96, *p* = 0.76, I2-0) as compared with open LIS. We recommended that closed lateral internal sphincterotomy (LIS) is a safe and effective surgical treatment option for chronic anal fissures.

## Introduction

Chronic anal fissure is one of the most common benign anorectal health conditions, causing significant morbidity, quality of life, and economic loss^1^. Evidence on epidemiological distribution has scared, but a population-based cohort in the United States reported that approximately 342,000 new anal fissure cases are diagnosed each year^1^. Anal fissure has an estimated average lifetime risk of 7.8%-11^2,3^. Approximately 90% of anal fissures are found in the posterior midline, 8–25% in the anterior midline, and 3% in the posterior and anterior positions of the anal canal^2,4^. The pathophysiological events underlying chronic anal fissure have been suggested to be attributed to mucosal injury and inflammation, leading to raised internal sphincter and local ischemia, which inhibits the process of healing^5^. While there are differences in the exact definition of a chronic anal fissure regarding to the duration, but the American Society of Colon and Rectal Surgeons (ASCRS) defines it as a fissure that lasts longer than six weeks with one or more stigmas of chronicity, such as a hypertrophied anal papilla at the proximal aspect of the fissure, a sentinel tag at the distal aspect of the fissure, and an exposed internal anal sphincter muscle at the base of the fissure^5^. The presenting complaint of chronic anal fissure is pain during defection, rectal bleeding, and emotional stress that it causes devastate quality of life. The treatment of chronic anal fissure has been done in a step-by-step fashion. The main goal of chronic anal fissure management is to reduce the pressure on the internal sphincter muscle using physical, chemical, and surgical methods to increase blood flow and decrease spasm of sphincter. Conservative measures have been considered to be the baseline of chronic anal fissure management^4^. Conservative treatment resolves approximately 90% of acute anal fissures. The American Society of Colon and Rectal Surgeons (ASCRS) recommends stool softeners, a high fiber diet, and a warm sitz bath for the initial nonsurgical management of anal fissure^4,5^. Evidence from meta-analysis and randomized controlled trials revealed that pharmacological agents or chemical sphincterotomy have been proposed as alternatives to surgery for the treatment of chronic anal fissure for last three decades^6–8^. However, when chronic fissures form, healing becomes more difficult, and only half of chronic anal fissure patients respond to conservative treatment^2,4^. Recent systemic reviews and randomized controlled trials investigate that surgical treatment of chronic fissures has a higher healing rate and improves quality of life when compared to conservative and pharmacological agents, despite the possibility of short- and long-term complications^9–23^. As a result, current guidelines from several international societies of colon and rectal surgeons recognize lateral internal sphincterotomy as the gold standard management option for chronic anal fissure^4,5,24,25^. Technically, an open lateral internal sphincterotomy was performed by exposing the lateral anal canal using retractor, making a 1 cm skin incision in the intersphincteric grove, separating the mucosa from the internal sphincter up to the dentate line, sphincter separated, and dividing the distal third of the sphincter with direct vision and securing hemostasis. Closed lateral internal sphincterotomy was performed by internal sphincter was palpated, 1 cm incision was made at intersphincteric grove, divided the distal one third internal sphincter with scalpel and hemostasis secured with direct pressured^26–28^.

Despite of the standard treatment for chronic anal fissures is laternal internal sphincterotomy; there is a paucity of high-quality evidence regarding the safety and efficacy of both closed and open laternal internal sphincterotomy. The primary goal of this review was to provide qualitive evidence on the efficacy and safety of closed and open lateral internal sphincterotomy in the treatment of chronic anal fissure.

## Results

**Figure 1:**
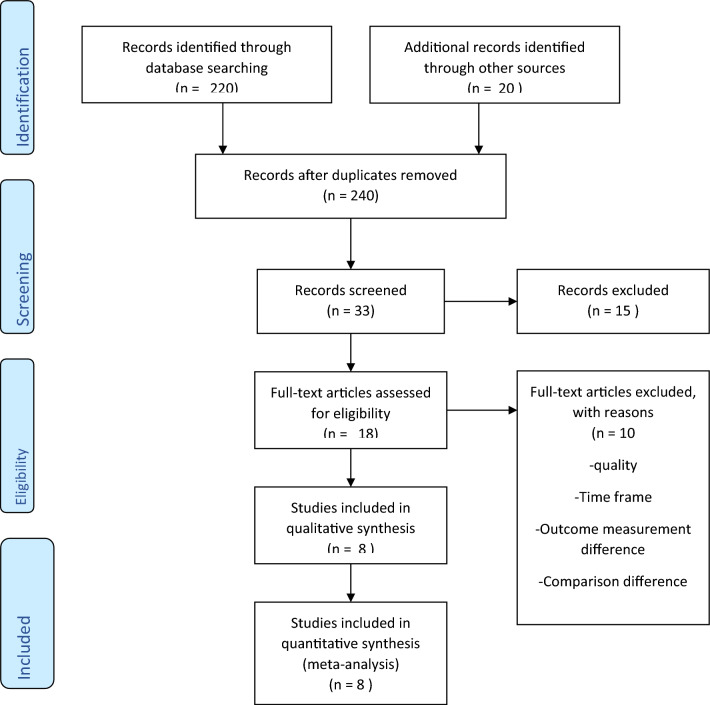
Flow of chart for selection of papers. Charts the selection of articles in the review in line with the Preferred Reporting Items for Systematic Review and Meta-analysis (PRISMA) Framework.

**Figure 2:**
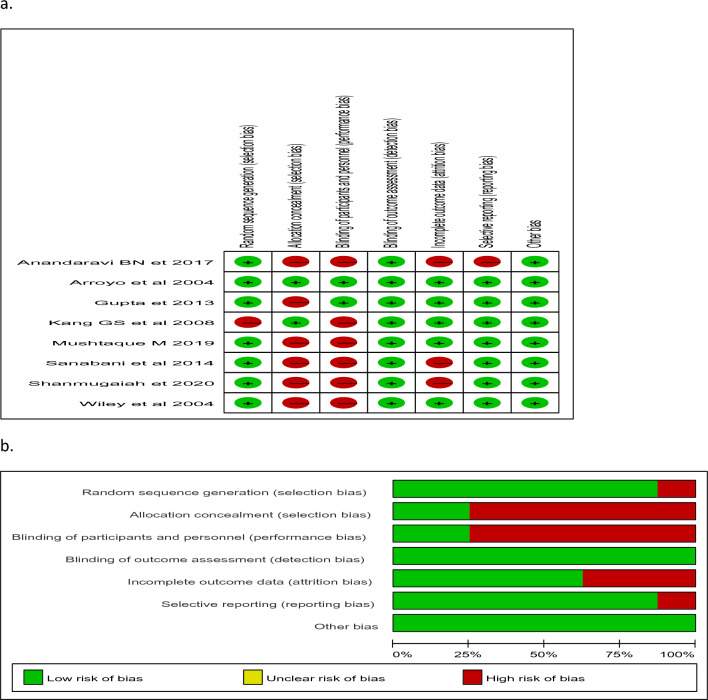
(**a**) and (**b**) Methodology quality of the included articles.

**Figure 3:**
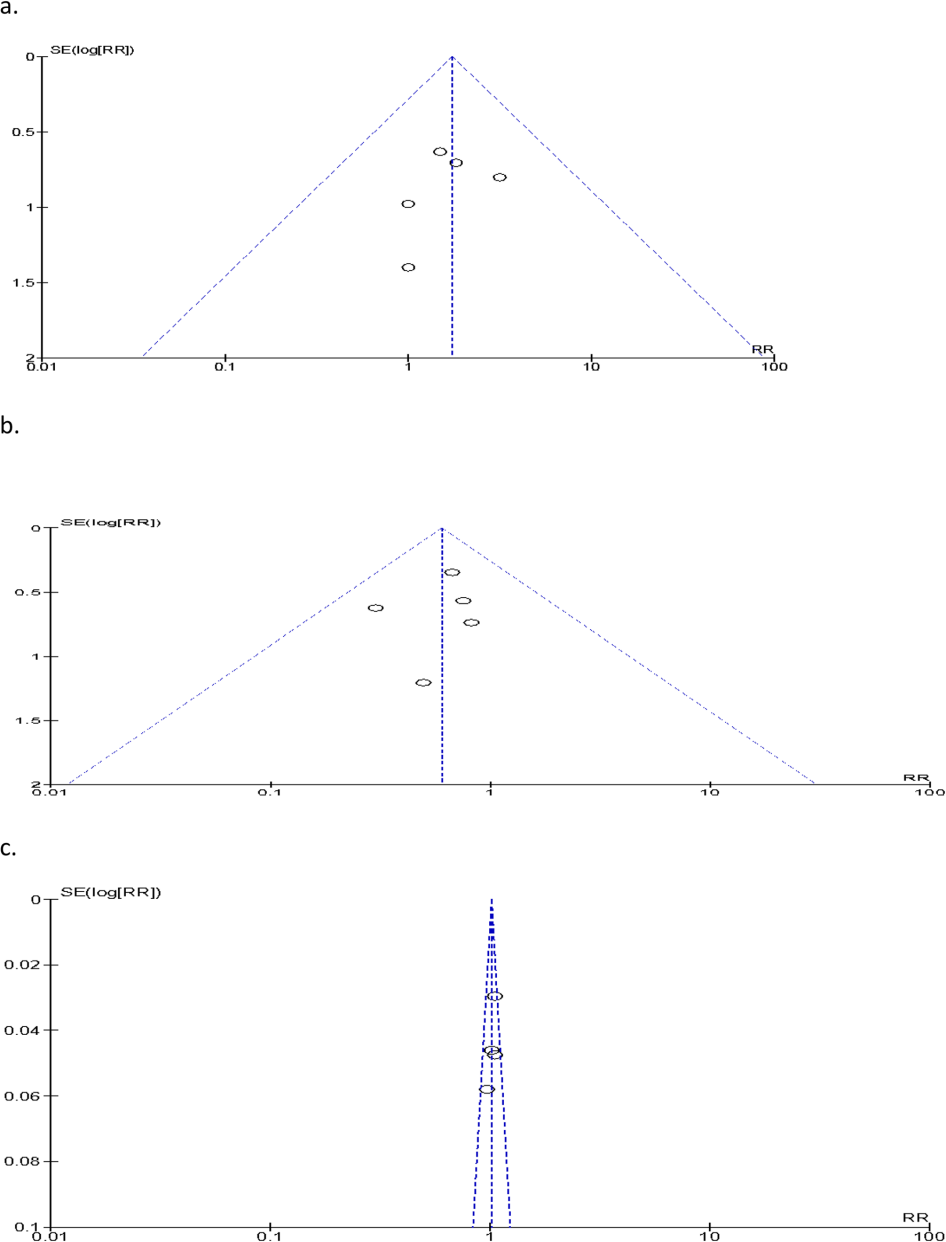
(**a**,**b)** and (**c**) Funnel plot of comparison of anal fissure, postoperative incontinent, and healing rate respectively.

We found 240 articles by searching an electronic database for key words and MeSH terms, hand searching using bibliographic or reference information from identified studies, and contacting authors to obtain online unavailable studies (Fig. 1). One article has low risk while seven article has high risk of biases. Full text assessments were performed on 18 articles, of which ten were excluded from the thesis due to study design^29–34^, difference in outcome measurement^35,36^, time frame, different comparison^37^, and poor quality^38^. Eight randomized controlled trials with a total population size of 1035 were eligible for analysis^26,27,39–44^. One article has a low risk of biases, while seven studies of the included articles have a high risk (Fig. 2a,b) however, there were no significant publication biases (Fig. 3a,b,c). Seven studies included both males and females^26,27,39,41–43^, while one only included females^40^. Majority participants in the included articles were females. After excluding study conducted on females only^40^, females were accounted 54.9% (43.5–66.3%, I2 = 92%, *p* = 0.001). The posterior chronic anal fissure was the most common location of the fissure, followed by the anterior location in all studies. The posterior chronic anal fissure was accounted 86.1% (95% CI 81.5–90.8%, I2 = 76.3%, *p* = 0.001).

### Recurrence rate

**Figure 4:**
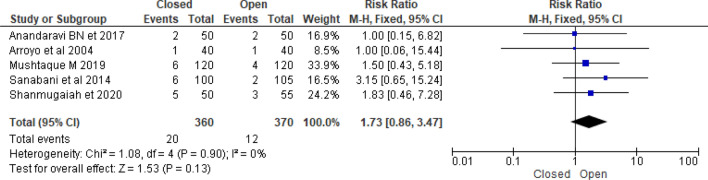
Forest plot display the comparison of recurrent anal fissure of Closed vs. Open LIS.

Five randomized controlled trials compared the rate of recurrence between closed and open lateral internal sphincterotomy for management of chronic anal fissure. According to Gupta et al.^39^, there was no recurrent anal fissure either the open or closed groups. Arroyo et al.^26^ investigated that the open sphincterotomy group had a 2.5% recurrent anal fissure and the closed sphincterotomy group had a 2.5% recurrent anal fissure^22^. According to Shanmugaiah and Pandian^27^, there was no significant difference in recurrent anal fissure between closed and open LIS^23^. However, Sanabani et al.^40^ reported that open LIS has lower recurrence anal fissure than closed LIS [6% versus 1.9% (*p* = 0.015)]. Mushtaque^44^ conducted a randomized controlled trial with 240 chronic anal fissures found that the recurrence of anal fissure was 3.3% in open LIS and 5% in closed LIS. Overall recurrence rate of fissure was 3.7% after LIS (2.3% in closed and 1.4% in open) (Table 1). Analysis of 730 patients from five randomized control trials noted that recurrent anal fissure rate was higher in closed as compared to open LIS [RR = 1.73 (95% CI 0.86–3.47, *p* = 0.13, I^2^ = 0%)], however, it was no statistical difference (Fig. 4).

**Table 1:** Characteristics of the included studies.

Authors	Country, Year	Study design	Sample close/open	Recurrent Anal Fissure		Post-operative incontinence		Healing		Post-operative pain		Post-operative bleeding		Infection	
				Closed	Open	Closed	Open	closed	Opened	Closed	Opened	Closed	Opened	Closed	Opened
Shanmugaiah et 2020	Indian	RCT	50 vs 55	5	3	3	4	–	–	8	20	5	1	2	10
Gupta et 2013	Indian	RCT	68vs 68	0	0	0	0	68/68	65/68	–	–	–	–	–	–
Sanabani et al. 2014	Yemen	RCT	100vs105	6	2	5	7	–	–	20	30	4	0	1	1
Anandaravi BN et 2017	Indian	RCT	50vs 50	2	2	3	10			1	6	2	8	1	3
Arroyo et al. 2004	Spain	RCT	40 vs 40	1	1	1	2					0	1		
Kang GS et al. 2008	South Korea	RCT	45 vs. 45					41/42	41/44						
Wiley et al. 2004	Australia	RCT	38 vs. 41					35/36	38/40						
Mushtaque M 2019	India	RCT	120vs 120	6	4	12	18	98/120	102/120			1	2

### Healing rate

**Figure 5:**
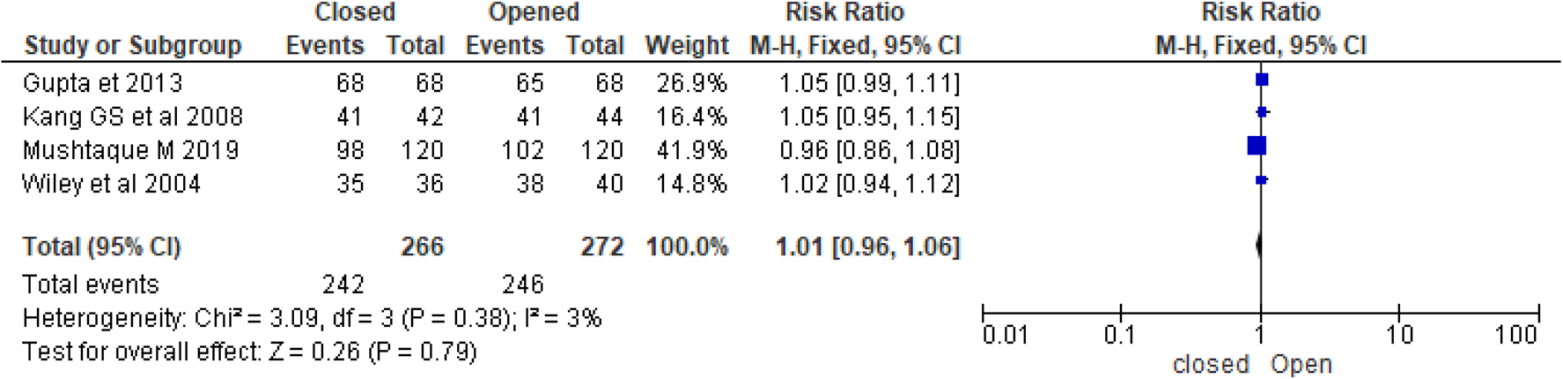
Forest plot display the comparison of healing rate between Closed vs. Open LIS.

In this systematic review, four randomised control trials that reported fairly similar healing rates following lateral internal sphincterotomy for management chronic anal fissure. The overall healing rate was 90.1% in the closed LIS and 90.3% in the open LIS, with overall healing rate after LIS was 90.2%. Gupta et al.^39^ conducted a prospective randomized comparative study reported that delayed healing was seen in 4.4% (*p* = 0.08) of open sphincterotomy patients and none of the patients in the closed sphincterotomy group had either delayed wound healing or an absence of wound healing postoperatively. According to Kang^42^, the chronic anal fissure-healing rate was 95% in closed and 93% in open, with the difference in healing rate between the two groups not statistically significant. Evidence from a randomised control trial conducted by Wiley^43^ revealed that the healing rate in closed LIS was 97% and 95% in open LIS. Mushtaque^44^ conducted a randomized controlled trial with 240 chronic anal fissures found that healing rate of fissure was 85% in opened and 81.6% in closed (Table 1). Analysis of 538 patients from four randomized control trials noted healing rate after LIS had no statistical difference between in closed and open LIS (RR = 1.0 (0.96, 1.06), *p* = 0.98, I^2^ = 3%) (Fig. 5).

### Incontinence rate

**Figure 6:**
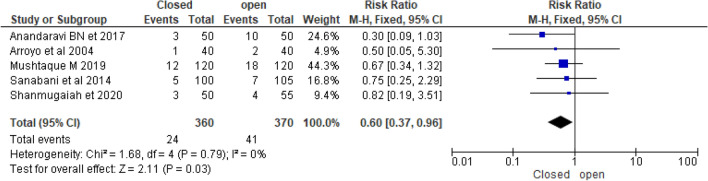
Forest plot display the comparison of post-operative incontinence rate between Closed vs. Open LIS.

Five randomized controlled trials compared the rate of incontinence between closed and open LIS. According to Gupta et al.^39^, there was no postoperative incontinence or soiling in either the open or closed groups. Arroyo et al.^26^ reported that the open sphincterotomy group had a 5% incontinence rate and the closed sphincterotomy group had a 2.5% incontinence rate (*p* 0.05). According to Anandaravi and Ramaswami^41^, 6% of closed LIS and 20% of open LIS were complicated with incontinence postoperatively. Overall postoperative incontinence rate after LIS was 8.9% (6.6% in closed and 11.0% in opened) (Table 1). Pooled postoperative incontinence rate was lowered in closed as compared to open [RR = 0.60 (95% CI 0.37–0.96, *p* = 0.0.03, I^2^-0%)] (Fig. 6).

### Postoperative bleeding

**Figure 7:**
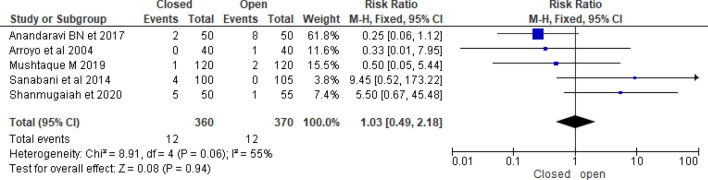
Forest plot display the comparison of postoperative bleeding between Closed vs. Open LIS.

Five randomized controlled trials compared postoperative bleeding between closed and open LIS. According to Gupta et al.^39^, there was no significant postoperative bleeding either the open or closed groups. Arroyo et al.^26^ investigated that the open sphincterotomy group had a 2.5% postoperative bleeding while closed sphincterotomy group had no postoperative bleeding. According to Shanmugaiah and Pandian^27^, there was significant difference in postoperative bleeding between closed and open LIS (4 vs.16%). However, Sanabani et al.^40^ reported that open LIS has lower recurrence anal fissure than closed LIS (0%vs.4%) (Table 1). Pooled postoperative bleeding rate was higher in closed as compared [RR = 1.0 (95% CI 0.49–2.18, *p* = 0.96, I^2^-59%)] (Fig. 7).

### Postoperative pain and infection

**Figure 8:**
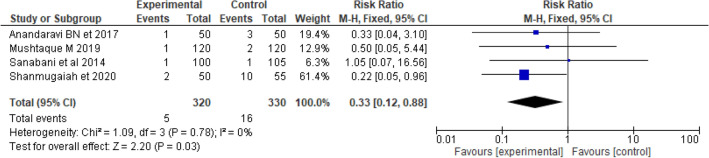
Forest plot display the comparison of postoperative infection between Closed vs. Open LIS.

**Figure 9:**
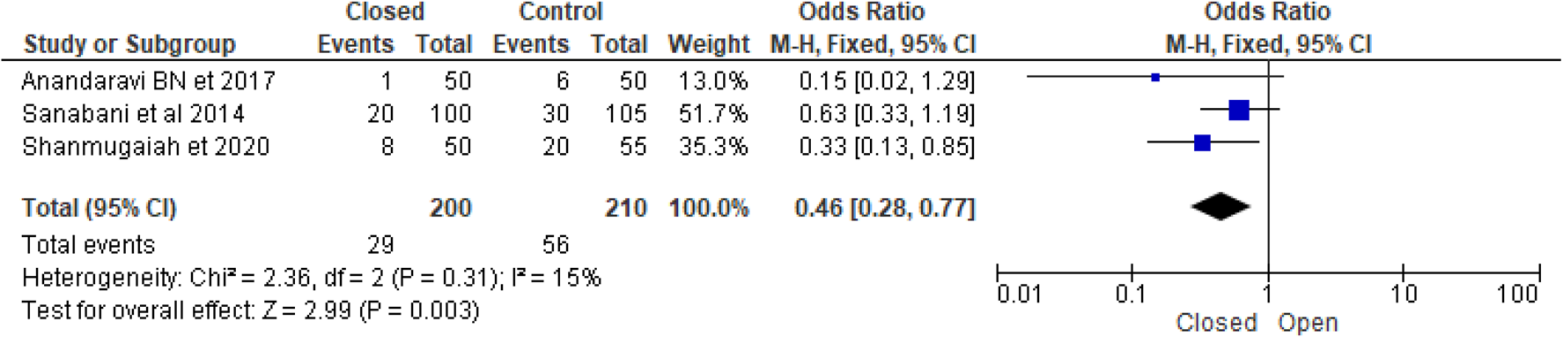
Forest plot display the comparison of postoperative pain between Closed vs. Open LIS.

Postoperative pain and infections are more common in open LIS. The postoperative infection rate was lesser in closed as compared to open LIS [RR = 0.29 (0.10, 0.80), *p* = 0.03, I^2^ = 0)]. The postoperative pain also lowered in closed as compared to open LIS [RR = 0.46 [0.28, 0.77), *p* = 0.003, I^2^ = 15)] (Figs. 8 and 9).

## Discussion

This study is a first meta-analysis of RCTs comparing the efficacy of closed and open Lateral internal sphincterotomy (LIS) in management of chronic anal fissure. Lateral internal sphincterotomy is a gold standard management option for chronic anal fissure, with consistently superior healing rates when compared to medical therapy^4,5,11^. The goal of lateral internal sphincterotomy is to lower resting anal tone due to internal anal sphincter which later increase the blood supply to anoderm to improve the healing.

This meta-analysis demonstrated that closed lateral internal sphincterotomy had a higher risk of recurrent anal fissure and a lower postoperative incontinence than open LIS, despite the differences not being statistically significant. In addition, there was no difference in postoperative bleeding and healing between closed and open LIS. Nevertheless, there was a statistically significant difference in postoperative pain and infection rates between closed and open LIS.

The result of this meta-analysis is consistent with a Cochrane analysis of five studies with 336 patients, which found no statistically significant difference between open and closed laternal internal sphincterotomy in terms of postoperative incontinence and healing rate^45^.

The main goal of chronic anal fissure management is to reduce the pressure on the internal sphincter muscle using physical, chemical, and surgical methods. Conservative measures such as a fiber diet, sitz baths, and stool softeners were considered to be the baseline of chronic anal fissure management^4,5,8^. The American Society of Colon and Rectal Surgeons (ASCRS) has been recommended conservative managements for the initial nonsurgical management of anal fissure^4,5^. Pharmacological agent or chemical sphincterotomy such as glyceryl trinitrate, calcium blockers, as well as botulinum toxin (BT) injection have been used as alternative of surgery for last three decades. However, recent systemic reviews and randomized controlled trials evidences showed that surgical treatment of chronic fissures has a higher healing rate and improves quality of life when compared to conservative and pharmacological agents^9–23^.

This meta-analysis found that LIS had a 90.2% success rate in treating chronic anal fissures (90.1% in closed and 90.3% in open). This findings were consistent with previous studies that reported an estimated success rate of 96%-100% for lateral internal sphincterotomy (LIS)^46,47^. However, these findings were higher when compared to the conserve management success rate of 65–75%^11,48,49^. When conservative medical treatments fail, lateral internal sphincterotomy (LIS) is considered the gold standard for the surgical management of chronic anal fissures^4^. However, approximately 3% of patients experience major wound-related complications that necessitate reoperations, such as bleeding, abscess or non-healing wound, and fistula^50^. Furthermore, it is important to remember that laternal internal sphincterotomy should not be done for obstetric trauma, documented anal sphincter injuries, or baseline faecal incontinence^51^. Patients may occasionally present with a triad syndrome such as a hypertrophied anal papilla at the proximal aspect of the fissure, a sentinel tag at the distal aspect of the fissure, and chronic anal fissure. Currently available low-quality comparative suggests excision of the skin tag and hypertrophied papilla along lateral internal sphincterotomy has been associated to reduced pain, irritation during defection, decreased foreign body sensation, and decreased pruritis^51,52^.

One of the most feared complications after a sphincterotomy is postoperative incontinence. Our meta-analysis of five RCT revealed that overall postoperative incontinence rate after LIS was 8.9% (6.6% in closed and 11.0% in opened). This finding was slightly lower than evidence obtained from a systemic review of 22 studies, which revealed that the overall continence disturbance rate after lateral internal sphincterotomy (LIS) for chronic anal fissure (CAF) was around 14%^53^. Various evidences suggested that the rate of postoperative incontinence differed between closed and open lateral internal sphincterotomy (LIS). Even though there was no statistically significant difference, our meta-analysis of randomised control trial studies revealed that the pooled postoperative incontinence rate was 40% lower in closed lateral internal sphincterotomy studies than in open lateral internal sphincterotomy. This finding is consistent with previous published comparative studies.

A low rate of recurrent anal fissure is one solid indicator to choose a specific type of intervention for chronic anal fissure. We found that overall recurrence rate of fissure was 3.7% after LIS (2.3% in closed and 1.4% in open). This finding was consistent with a previous study that found that the rate of recurrent anal fissure was 3.6% in 417 chronic anal fissure operated patients with an eight-year follow-up^47^. Evidence on the efficacy of closed and open LIS in the prevention of recurrent anal fissure has been scarred. This meta-analysis of randomized controlled trial studies found that closed LIS had approximately two-fold increased risk of developing recurrent anal fissures when compared to open LIS, but this was not statistically significant. A closed LIS reduces the risk of post-operative infection, which has a significant morbidity and hospital stay burden.

This meta-analysis has some limitation. Some of the included articles have small sample sizes, imprecise reporting of concealment, and blinding techniques. In the included studies, various scales and instruments of measurement have been used for some outcomes, like the pain score which would affect the pooled estimation.

Conclusion: There is no statistically significant difference in recurrent anal fissure, postoperative bleeding, and postoperative healing rate between closed and open LIS. Acute LIS complications such as postoperative incontinence, postoperative infection, and pain are less common in closed LIS. We recommended that closed LIS is a safe and effective surgical treatment option for chronic anal fissures.

## Methods

### Study design

We used a systematic review and meta-analysis study design to summarize randomised control trial studies published between January 1, 2000 and December 30, 2022. We reported the findings in accordance with the Protocol for Preferred Reporting Items for Systematic Reviews and Meta-analyses (PRISMA)^54,55^. The protocol registered in PROSPERO website (CRD42023392626).

### Eligibility criteria

Randomized controlled trials studies published between January 1, 2000 and December 30, 2022 on patients with chronic anal fissure who underwent either closed or open lateral internal sphincterotomy were considered to be eligible for inclusion. Systematic review studies, observational studies, cohort studies, and studies with unclearly reported results were excluded from the analysis. The English language restriction and time frame were chosen for convenience and sufficiency for demonstrating a trend of events. Eligible articles were identified using key words and MeSH words in an electronic database, hand searching using bibliographic or reference information from identified studies, and contacting authors to obtain online unavailable studies^56,57^.

### Searching strategies, searching sources and selection

We searched Medline, CINAHAL, Web Science, Google Scholar, the Cochrane Library, and ClinicalTrials.gov for eligible articles. Two separate reviewers conducted the electronic data base search and selection eligible articles. Disagreements between two reviewers are resolved through discussion, consensus, and, if necessary, the involvement of a third party. Our searching strategy was based on the Population, Interventions, Comparison, Outcomes, and Study (PICOS) approach^54^. Searching was done using free text and MeSH words. Boolean operator, Wild cards, and splinting words and phrases were employed to widen of our search^56–60^. We used ‘’Chronic anal fissure’’, ‘’Closed lateral internal sphincterotomy’’, ‘’open lateral internal sphincterotomy’’, ‘’anal fissure’’, ‘’randomised control trial’’, ‘’RCT’’, ‘’lateral internal sphincterotomy’’ to locate the eligible articles in different electronic data base.

Two authors independently screened using inclusion criteria. Duplicate studies from various electronic databases obtained through the search strategy were removed using the EndNote program. The titles and abstracts of the articles found through the search strategy were independently screened by two reviewers to eliminate obvious non-relevant papers. The full-text versions of the remaining potentially eligible articles were then retrieved and independently assessed by two reviewers to determine if they met the inclusion criteria. Disagreements are resolved through consensus and discussion. The corresponding author was contacted in order to obtain copies of papers whose full text was not available online. The reasons for exclusion at the full-text screen level were documented in accordance with the PRISMA framework. The total number of unique studies from all sources that meet the inclusion criteria that have been recorded^60^ (Fig. 7).

### Data extraction and management

Data were extracted using a piloted standardized data extraction form adapted and customized from the Cochrane data extraction of randomised and non-randomised studies. The publication details, language of the paper, study period, study location, geographic setting, study design, study period, characteristics of participants, sample size and sampling technique, explanatory and outcome variables, data analysis, and the major findings were extracted.

### Risk of bias assessment

The included studies' quality was assessed using RoB2 assessing tools that were specifically designed and validated for RCT studies^61^. The RoB2 quality assessment tool has seven items to asses in each trial include random sequence, allocation concealment, blindness of participants, blindness of outcome assessment, outcome data incompleteness, selecting reporting, and other biases. Two reviewers independently evaluated the quality of the included papers and characterize them as having a high, some concern or low risk of bias.

### Assessment of heterogeneity

Heterogeneity is a deviation from the true effect in meta-analysis caused by chance or variability in the included studies. The sources of heterogeneity would be clinical, methodological, and/or statistical. The I square statistic (I^2^) represents the percentage of variability in effect estimates caused by heterogeneity^62^. Consider I^2^ values of 25%, 50%, and 75% to have low, moderate, or high heterogeneity^63,64^. Clinical, methodological and/or statistical heterogeneity assessed and method for addressing the heterogeneity such as sensitivity analysis, meta-regression, and subgroups analysis documented^64^.

### Publication biases

Publication bias is a deviation from true or standard value caused by a deviated result or processes. Bias results from deviations from the standard norm in data collection, analysis, interpretation, review, and/or publication^64,65^. Funnel plot asymmetry (test for publication bias) investigates the association between the effect size estimate and measure of study size or precision. It shows the distribution of included studies in a meta-analysis against a measure of size of effect or precision (i.e. standard error)^62^. Largest studies in the meta-analysis should be closes to true value whereas the smallest studies spread on either side; creates a funnel shape. Symmetry funnel shape has been seen when no publication bias whereas asymmetry or skewed funnel shape observed in publication bias^64^.

### Data analysis

The meta-analysis performed using Review Manager version 5.4(Rev5.4). The comparison of clinical and outcomes of chronic anal fissure was conducted between closed and open LIS. The pooled analysis was reported with risk ratio (RR) with 95% confidence interval for categorical data while mean difference (MD) with 95% confidence interval for continuous variables. The mean and standard deviations (SD) were computed using accepted methods from the available median, interquartile range, and confidence interval or range if the result was reported with a median and an interquartile range. Fixed -effect model using Mantle-Haenszel method was employed.

## Data Availability

The datasets used and/or analysed during the current study available from the corresponding author on reasonable request.

## Abbreviations

CAF: Chronic anal fissure
LIS: Lateral internal sphincterotomy
PICOS: Population, intervention, comparison, outcomes, study design
RCT: Randomised control trial

## References

[1] Mapel DW, Schum M, Von Worley A (2014). The epidemiology and treatment of anal fissures in a population-based cohort. BMC Gastroenterol..

[2] Salati SA (2021). Anal fissure—An extensive update. Pol. Przegl. Chir..

[3] Cross KL, Massey EJ, Fowler AL, Monson JR (2008). ACPGBI. The management of anal fissure: ACPGBI position statement. Colorectal Dis..

[4] Stewart DB, Gaertner W, Glasgow S, Migaly J, Feingold D, Steele SR (2017). Clinical practice guideline for the management of anal fissures. Dis. Colon. Rectum..

[5] Davids, J. S. *et al.*; Clinical Practice Guidelines Committee of the American Society of Colon and Rectal Surgeons. The American Society of Colon and Rectal Surgeons Clinical Practice Guidelines for the Management of Anal Fissures. *Dis. Colon Rectum.* **66**(2), 190–199. 10.1097/DCR.0000000000002664 (2023). 10.1097/DCR.0000000000002664 36321851

[6] Sahebally SM, Meshkat B, Walsh SR, Beddy D (2018). Botulinum toxin injection vs topical nitrates for chronic anal fissure: An updated systematic review and meta-analysis of randomized controlled trials. Colorectal Dis..

[7] Lin JX, Krishna S, Su'a B, Hill AG (2016). Optimal dosing of botulinum toxin for treatment of chronic anal fissure: A systematic review and meta-analysis. Dis. Colon. Rectum..

[8] Beaty JS, Shashidharan M (2016). Anal fissure. Clin. Colon. Rectal. Surg..

[9] Acar T, Acar N, Güngör F, Kamer E, Genç H, Atahan K, Dilek ON, Hacıyanlı M (2020). Comparative efficacy of medical treatment versus surgical sphincterotomy in the treatment of chronic anal fissure. Niger. J. Clin. Pract..

[10] Ahmad MS, Amin I, Kareemullah M, Bashir S, Sattar Z, Hanif A (2014). Outcome of botulinum toxin with lateral internal sphincterotomy for treatment of chronic anal fissure. Pak. J. Med. Health Sci..

[11] Nelson RL, Manuel D, Gumienny C, Spencer B, Patel K, Schmitt K, Castillo D, Bravo A, Yeboah-Sampong A (2017). A systematic review and meta-analysis of the treatment of anal fissure. Tech. Coloproctol..

[12] Algaithy ZK (2008). Botulinum toxin versus surgical sphincterotomy in females with chronic anal fissure. Saudi Med. J..

[13] Arslan K, Erenoğlu B, Doğru O, Turan E, Eryilmaz MA, Atay A, Kökçam S (2013). Lateral internal sphincterotomy versus 0.25% isosorbide dinitrate ointment for chronic anal fissures: A prospective randomized controlled trial. Surg. Today..

[14] Aslam MI, Pervaiz A, Figueiredo R (2014). Internal sphincterotomy versus topical nitroglycerin ointment for chronic anal fissure. Asian J. Surg..

[15] Bokhari ST, Zubair M, Rasheed S, Shaukat H, Munir MS (2020). Arujalam outcome of chemical and surgical lateral internal sphincterotomy for acute anal fissure. Pak. J. Med. Health Sci..

[16] Brown CJ, Dubreuil D, Santoro L, Liu M, O'Connor BI, McLeod RS (2007). Lateral internal sphincterotomy is superior to topical nitroglycerin for healing chronic anal fissure and does not compromise long-term fecal continence: Six-year follow-up of a multicenter, randomized, controlled trial. Dis. Colon. Rectum..

[17] Butt F, Aslam MN, Nadeem N (2017). Comparison of lateral internal sphincterotomy and GTN gel for management of chronic anal fissure: A randomized controlled trial. Pak. J. Med. Health Sci..

[18] de Rosa M, Cestaro G, Vitiello C, Massa S, Gentile M (2013). Conservative versus surgical treatment for chronic anal idiopathic fissure: A prospective randomized trial. Updates Surg..

[19] Giridhar CM, Babu P, Rao KS (2014). A comparative study of lateral sphincterotomy and 2% diltiazem gel local application in the treatment of chronic fissure in ANO. J. Clin. Diagn. Res..

[20] Ibrahim, S., Natarajan, R., Mohamed Zakkariya, A. R., Loganathan, M. A comparative study of topical 2% diltiazem with lateral sphincterotomy in the treatment of chronic fissure in-ano. *Ann. Trop. Med. Public Health.* **23** (2023).

[21] Iswariah H, Stephens J, Rieger N, Rodda D, Hewett P (2005). Randomized prospective controlled trial of lateral internal sphincterotomy versus injection of botulinum toxin for the treatment of idiopathic fissure in ano. ANZ J. Surg..

[22] Motie MR, Hashemi P (2016). Chronic anal fissure: A comparative study of medical treatment versus surgical sphincterotomy. Acta Med. Iran..

[23] Jin JZ, Bhat S, Park B, Hardy MO, Unasa H, Mauiliu-Wallis M, Hill AG (2022). A systematic review and network meta-analysis comparing treatments for anal fissure. Surgery..

[24] Nelson, R. L. Operative procedures for fissure in ANO. *Cochrane Database Syst. Rev.* **20**(1), CD002199 (2010). 10.1002/14651858.CD002199.pub3. Update in: Cochrane Database Syst Rev. 2011;(11):CD002199. 10.1002/14651858.CD002199.pub4 PMC7098462 22071803

[25] Ebinger SM, Hardt J, Warschkow R, Schmied BM, Herold A, Post S, Marti L (2017). Operative and medical treatment of chronic anal fissures-a review and network meta-analysis of randomized controlled trials. J. Gastroenterol..

[26] Arroyo A, Pérez F, Serrano P, Candela F, Calpena R (2004). Open versus closed lateral sphincterotomy performed as an outpatient procedure under local anesthesia for chronic anal fissure: Prospective randomized study of clinical and manometric longterm results. J. Am. Coll. Surg..

[27] Shanmugaiah A, Pandian S (2020). Prospective randomized study between open vs closed lateral anal internal sphincterotomy in patients with chronic fissure in Ano. Acad. J Surg..

[28] Lu Y, Kwaan MR, Lin AY (2021). Diagnosis and treatment of anal fissures in 2021. JAMA..

[29] Mahabub M (2018). A comparison between the results of open versus closed lateral internal sphincterotomy in the surgical management of chronic anal fissure. BIRDEM Med. J..

[30] Sarhan HH (2012). Closed versus open lateral internal sphincterotomy in treatment of chronic anal fissure: A comparative study. Arch. Clin. Exp. Surg..

[31] Seleem A, Abbas M (2022). Comparative study between closed versus open internal sphincterotomy for management of chronic anal fissure. Egypt. J. Hos. Med..

[32] Sanniyasi S, Alexander N, Thiyagarajan M (2016). Open versus closed lateral internal sphincterotomy in chronic anal fissures: A prospective study. Int J Sci Stud.

[33] Mukri HM, Neeti Kapur N, Guglani V (2019). Comparison of open versus closed lateral internal sphincterotomy in the management of chronic anal fissure. Hellenic J. Surg..

[34] Garcia-Aguilar J, Belmonte C, Wong WD, Lowry AC, Madoff RD (1996). Open vs. closed sphincterotomy for chronic anal fissure: Long-term results. Dis. Colon. Rectum..

[35] Ahmed F, Azam Mengal M, Ahmed M (2018). Comparison of complications of open versus closed lateral internal sphincterotomy in chronic anal fissures. PJMHS.

[36] Hyman N (2004). Incontinence after lateral internal sphincterotomy: A prospective study and quality of life assessment. Dis. Colon. Rectum..

[37] Kortbeek JB, Langevin JM, Khoo RE, Heine JA (1992). Chronic fissure-in-ano: a randomized study comparing open and subcutaneous lateral internal sphincterotomy. Dis. Colon. Rectum..

[38] Bwelle Motto G, Ekani Boukar Y, Bang G, Ngoumfe J, Tientcheu Tim F, Essomba A, Sosso M (2021). Internal lateral sphincterotomy in yaounde: Comparative short-term results of open versus closed techniques. Surg. Sci..

[39] Gupta V, Rodrigues G, Prabhu R, Ravi C (2014). Open versus closed lateral internal anal sphincterotomy in the management of chronic anal fissures: A prospective randomized study. Asian J. Surg..

[40] Al Sanabani J, Al Salami S, Al Saadi A (2014). Closed versus open lateral internal anal sphincterotomy for chronic anal fissure in female patients. Egypt. J. Surg..

[41] Anandaravi BN, Ramaswami B (2017). Closed versus open lateral internal anal sphincterotomy in a chronic anal fissure. Int. Surg. J..

[42] Kang GS, Kim BS, Choi PS, Kang DW (2008). Evaluation of healing and complications after lateral internal sphincterotomy for chronic anal fissure: Marginal suture of incision vs. open left incision: prospective, randomized, controlled study. Dis. Colon. Rectum..

[43] Wiley M, Day P, Rieger N, Stephens J, Moore J (2004). Open vs. closed lateral internal sphincterotomy for idiopathic fissure-in-ano: A prospective, randomized, controlled trial. Dis. Colon. Rectum..

[44] Mushtaque, M. Lateral internal sphincterotomy in chronic anal fissures: a comparative study between open and closed techniques. *Int. J. Sci. Res.* **8**(3) (2019).

[45] Nelson RL, Chattopadhyay A, Brooks W, Platt I, Paavana T, Earl S (2011). Operative procedures for fissure in ano. Cochrane Database Syst. Rev..

[46] Liang J, Church JM (2015). Lateral internal sphincterotomy for surgically recurrent chronic anal fissure. Am. J. Surg..

[47] Acar T, Acar N, Güngör F, Kamer E, Güngör H, Candan MS, Bağ H, Tarcan E, Dilek ON, Haciyanli M (2019). Treatment of chronic anal fissure: Is open lateral internal sphincterotomy (LIS) a safe and adequate option?. Asian J. Surg..

[48] Pilkington SA, Bhome R, Welch RE, Ku F, Warden C, Harris S, Hicks J, Richardson C, Dudding TC, Knight JS, King AT, Mirnezami AH, Beck NE, Nichols PH, Nugent KP (2018). Bilateral versus unilateral botulinum toxin injections for chronic anal fissure: A randomised trial. Tech. Coloproctol..

[49] Chen HL, Woo XB, Wang HS, Lin YJ, Luo HX, Chen YH, Chen CQ, Peng JS (2014). Botulinum toxin injection versus lateral internal sphincterotomy for chronic anal fissure: A meta-analysis of randomized control trials. Tech. Coloproctol..

[50] Walker WA, Rothenberger DA, Goldberg SM (1985). Morbidity of internal sphincterotomy for anal fissure and stenosis. Dis. Colon. Rectum..

[51] Brillantino, A. *et al.* The Italian Unitary Society of Colon-proctology (SIUCP: Società Italiana Unitaria di Colonproctologia) guidelines for the management of anal fissure. *BMC Surg.* **23**(1), 311. 10.1186/s12893-023-02223-z (2023). 10.1186/s12893-023-02223-z PMC10576345 37833715

[52] Gupta PJ (2004). Hypertrophied anal papillae and fibrous anal polyps, should they be removed during anal fissure surgery?. World J. Gastroenterol..

[53] Garg P, Garg M, Menon GR (2013). Long-term continence disturbance after lateral internal sphincterotomy for chronic anal fissure: A systematic review and meta-analysis. Colorectal Dis..

[54] Moher, D. *et al.*; PRISMA-P Group. Preferred reporting items for systematic review and meta-analysis protocols (PRISMA-P) 2015 statement. *Syst. Rev.* **4**(1), 1. 10.1186/2046-4053-4-1 (2015). 10.1186/2046-4053-4-1 PMC4320440 25554246

[55] Shamseer, L. *et al.*; PRISMA-P Group. Preferred reporting items for systematic review and meta-analysis protocols (PRISMA-P) 2015: elaboration and explanation. *BMJ* **350**,g7647. 10.1136/bmj.g7647. Erratum in: BMJ. 2016 Jul 21;354:i4086. PMID: 25555855 (2015). 10.1136/bmj.g7647 25555855

[56] Edoardo Aromataris, E., Riitano, D. Constructing a search strategy and searching for evidence. *AJN* **114**(5) (2014). 10.1097/01.NAJ.0000446779.99522.f6 24759479

[57] McKenzie JE, Brennan SE, Ryan RE, Thomson HJ, Johnston RV, Thomas J, Higgins JPT, Thomas J, Chandler J, Cumpston M, Li T, Page MJ, Welch VA (2019). Chapter 3: Defining the criteria for including studies and how they will be grouped for the synthesis. Cochrane Handbook for Systematic Reviews of Interventions.

[58] Stern C, Jordan Z, McArthur A (2014). Developing the review question and inclusion criteria. Am J Nurs..

[59] Thomas J, Kneale D, McKenzie JE, Brennan SE, Bhaumik S, Higgins JPT, Thomas J, Chandler J, Cumpston M, Li T, Page MJ, Welch VA (2019). Chapter 2: Determining the scope of the review and the questions it will address. Cochrane Handbook for Systematic Reviews of Interventions.

[60] Lefebvre C, Glanville J, Briscoe S, Littlewood A, Marshall C, Metzendorf M-I, Noel-Storr A, Rader T, Shokraneh F, Thomas J, Wieland LS, Higgins JPT, Thomas J, Chandler J, Cumpston M, Li T, Page MJ, Welch VA (2019). Chapter 4: Searching for and selecting studies. Cochrane Handbook for Systematic Reviews of Interventions.

[61] Sterne JAC, Savović J, Page MJ, Elbers RG, Blencowe NS, Boutron I, Cates CJ, Cheng HY, Corbett MS, Eldridge SM, Emberson JR, Hernán MA, Hopewell S, Hróbjartsson A, Junqueira DR, Jüni P, Kirkham JJ, Lasserson T, Li T, McAleenan A, Reeves BC, Shepperd S, Shrier I, Stewart LA, Tilling K, White IR, Whiting PF, Higgins JPT (2019). RoB 2: A revised tool for assessing risk of bias in randomised trials. BMJ..

[62] Deeks, J. J., Higgins, J. P. T., Altman, D. G. (editors). Chapter 9: Analysing data and undertaking meta-analyses. In: Higgins JPT, Green S (editors). Cochrane Handbook for Systematic Reviews of Interventions. Wiley (2008).

[63] Higgins JPT, Thompson SG, Deeks JJ, Altman DG (2003). Measuring inconsistency in metaanalyses. BMJ.

[64] Tufanaru C, Munn Z, Aromataris E, Campbell J, Hopp L. Chapter 3: Systematic reviews of effectiveness. In: Aromataris E, Munn Z (Editors). JBI Manual for Evidence Synthesis. JBI, Available from https://synthesismanual.jbi.global. 10.46658/JBIMES-20-04 (2020).

[65] Song, F., Eastwood A, Gilbody S, Duley L, Sutton A. Publication and related biases: A review. *Health Technol. Assess.* **4**(10) (2000). 10932019

